# Impact of the Covid-19 Pandemic on Prostate Cancer Investigation and Diagnosis in the Brazilian Public Health System (SUS)

**DOI:** 10.1200/GO.22.60000

**Published:** 2022-05-05

**Authors:** Lucas Kieling, Izadora Bouzeid Estacia da Silveira, Luiza Seixas de Sá Beltramo, Yasmin Ricarte Hass Lopes, Gabriele Eckerdt Lech, Laura Tibola Marques da Silva, Danna Gomes Mateus

**Affiliations:** ^1^ Universidade Federal de Ciências da Saúde de Porto Alegre (UFCSPA), Porto Alegre, Brazil; ^2^ Pontifícia Universidade Católica do Rio Grande do Sul (PUCRS), Porto Alegre, Brazil

## Abstract

Prostate cancer was the second most deadly type of cancer in males in Brazil in 2019. Therefore, early diagnosis is fundamental to reduce its morbimortality. Diagnosis is essentially confirmed by prostate biopsy. The overload on health systems caused by the coronavirus pandemic has strongly affected the investigation process in Brazilian public healthcare (SUS). Under these circumstances, we aim to expose the effects of COVID-19 in the screening and diagnosis of prostate cancer performed by SUS.

**METHODS:**

A cross-sectional design of data collected from DATASUS's Hospital Information System. The study analyzes absolute numbers of prostate biopsies and diagnosis of prostate cancer registered between January and July in 10 years (2011-2020) which were comparatively evaluated. Statistical analysis was performed to assess the number of procedures compared over the years.

**RESULTS:**

The number of prostate biopsies in Brazil, through the Public Health System (SUS), between January 2011 and December 2019, was 393,678 with a yearly average of 43,742 procedures. In 2020, there was a 27.23% decrease, meaning only 31,829 procedures. In agreement with that, a decrease of 49.71%, 33.73%, 37.25% and 40.76% on prostate cancer diagnosis was reported in the months of April, May, June and July, respectively, when comparing 2019 and 2020. The total number of prostate cancer diagnoses also suffered a decrease of 27.32% between the years of 2019 and 2020, going from 41,981 procedures to only 30,511.

**CONCLUSION:**

The reduction on the number of prostate cancer diagnosis is evident and is going to affect the morbimortality of the patients who haven't had access to the health system during the covid 19 pandemic. It is fundamental to know the amount of damming on those diagnoses to propose measures to prevent the worst outcomes, reducing avoidable deaths.

